# Determining the factors influencing the selection of post-acute care models by patients and their families: a qualitative content analysis

**DOI:** 10.1186/s12877-023-03889-z

**Published:** 2023-03-28

**Authors:** Ya-Hui Hsu, Tsong-Hai Lee, Kuo-Piao Chung, Yu-Chi Tung

**Affiliations:** 1 Institute of Health Policy and Management, College of Public Health, National Taiwan University, Taipei, Taiwan; 2 Department of Anesthesiology, Linkou Chang Gung Memorial Hospital, Taoyuan, Taiwan; 3 Stroke Center, Department of Neurology, Linkou Chang Gung Memorial Hospital, Taoyuan, Taiwan; 4 College of Medicine, Chang Gung University, Taoyuan, Taiwan

**Keywords:** Stroke, Post-acute care model, Patient-centred care, Functional disability, Rehabilitation

## Abstract

**Background:**

This study conducted in-depth interviews to explore the factors that influence the choice of a post-acute care (PAC) model (inpatient rehabilitation hospital, skilled nursing facility, home health, and outpatient rehabilitation) among stroke patients and their families.

**Methods:**

We conducted semi-structured, in-depth interviews of 21 stroke patients and their families at four hospitals in Taiwan. Content analysis was used in this qualitative study.

**Results:**

Results revealed five main factors that influence respondents’ choice of PAC: (1) medical professionals’ suggestions, (2) health care accessibility, (3) continuity and coordination of care, (4) willingness and prior experience of patients and their relatives and friends, and (5) economic factors.

**Conclusions:**

This study identifies five main factors that affect the choice of PAC models among stroke patients and their families. We suggest that policymakers establish comprehensive health care resources based on the needs of patients and families. Health care providers shall provide professional recommendations and adequate information to support decision-making, which aligns with the preferences and values of patients and their families. From this research, we hope to improve the accessibility of PAC services in order to enhance the quality of care for stroke patients.

## Introduction

Stroke is the second leading cause of death in the world, accounting for approximately 6.2 million deaths worldwide each year [1, 2], and it is also the leading cause of long-term disability in adults [3]. Studies have shown that one-third of haemorrhagic stroke patients had functional dependence or died within three months after the stroke episode [4]. For surviving stroke patients, functional disability is a crucial issue. Following acute treatment and stabilisation of vital signs, follow-up rehabilitation therapy is the key to restoring the lost functions [5, 6].

The concept of post-acute care (PAC) refers to follow-up care for discharged patients, which means that even after receiving acute hospital treatment, patients still require complete health care services to recover their original body functions. The aim of PAC is to minimise patients’ physical disability to enable them to return to community life smoothly [7, 8]. In order to cope with an ageing population and resolve the potential burden on the health care system, family, and social care providers due to the possibility of an increasing number of patients suffering from disabilities after acute treatment, in Taiwan, the National Health Insurance Administration not only reimburses post-acute inpatient and outpatient rehabilitation as well as home care, but also has implemented the Integrated Post-Acute Care Plan for stoke to improve the quality of post-acute care since 2014. The plan assigned active integrated care for stroke patients during the golden rehabilitation period after discharge from acute care hospitalisation, including inpatient rehabilitation and home rehabilitation, based on the patients’ disability level and the assessment results of the PAC team in order to improve the quality of medical services and the efficiency of the use of medical resources through the reform of the payment system [9]. Therefore, in Taiwan, the PAC models include inpatient rehabilitation hospital, skilled nursing facility, home health, and outpatient rehabilitation.

Previous studies on PAC and care outcomes have found that PAC reduced mortality risk [10, 11], promoted functional recovery, helped stroke patients return to their home and communities, and improved the quality of life of stroke patients [12]. The different PAC models for stroke patients (including inpatient rehabilitation hospital, skilled nursing facility, home health, and outpatient rehabilitation) also affected the mortality risk within one year [11], indicating the importance of PAC and the effect of the choice of PAC models on the quality of care for stroke patients. Another study on inpatients over the age of 55 in the United States who were recommended to receive PAC services found that approximately 30% of these patients refused PAC services, and that the risk of readmission within 30 days among these patients was twice that of patients receiving PAC [13]. In summary, although PAC services produce actual benefits on patient care outcomes, patients and their families may not necessarily accept PAC services as recommended by professional medical teams. Patients and their families have more influence than clinicians in choosing the appropriate PAC models [14]. Regarding this issue, the practice followed by the Centers for Medicare and Medicaid Services (CMS) may be used as reference; it recommends that medical providers discuss post-discharge PAC plans with patients and their families, and that patients be encouraged to decide on PAC services to improve patient care outcomes [15]. Therefore, it is important to determine the factors influencing the acceptance of PAC by patients and their families as well as the choice of different PAC models. The results will help both doctors and patients communicate regarding the choice of PAC services.

Generally speaking, after a patient is discharged from acute inpatient care, the clinical medical team would decide on or recommend a PAC model to the patient based on their main diagnosis, comorbidities, disability, and cognitive functions. Factors influencing the acceptance of certain PAC models or the rejection of PAC services by patients are worth exploring. Previous studies on factors affecting patients’ choice of PAC models mostly used medical data, including medical insurance and related data, as research data sources, and classified the reasons into four possible categories, namely financial, personal, structural, and attitudinal barriers [16]. Few studies have explored the factors affecting the patients’ choice of PAC models from a patient- and family-centred perspective. There were two qualitative studies on the experiences of patients receiving outpatient rehabilitation [17] and inpatient rehabilitation [18] during the post-acute stage. One article investigated the experience of patients and family members regarding the planning of discharge and the process of choosing a skilled nursing facility model [19], while the other article investigated the reasons why patients requiring PAC refused PAC services. However, the latter study selected study subjects over the age of 55 [20]. There has been no study discussing the factors influencing the selection of PAC models among stroke patients and their families.

Therefore, this study explored the factors influencing the choice of PAC services and models among stroke patients and their families through qualitative in-depth interviews. The findings of this study may serve as a reference for policy makers and medical providers on the requirements and goals for the post-discharge care of stroke patients.

## Methods

### Study design

This study was a qualitative study using a semi-structured interview outline and in-depth interview records as the content of data analysis. In-depth interview is one of the widely-used methods in qualitative research [21, 22]. The semi-structured interview outline provided a specific, yet flexible and open-ended question framework for the topic to be explored [23]. Therefore, a semi-structured interview outline (see Table 1) was developed according to the research purpose, and the researchers conducted face-to-face interviews with stroke patients and their families based on the this outline, hoping to help the interviewees reveal their motivations and ideas behind choosing PAC services and models.

**Table 1:** Outline of semi-structured interview on factors influencing the choice of post-acute care models for stroke patients and their families

1. Can you tell me about your hospitalisation first? Why are you here?
2. How long after you were admitted to the hospital did someone tell you that you might need to continue treatment and rehabilitation elsewhere after being discharged?
3. Do you or your family know where you can receive post-acute care and rehabilitation after discharge? (Inpatient rehabilitation hospital, skilled nursing facility, home health, and outpatient rehabilitation)? Where did you get this information? Has anyone discussed it with you?
4. Why did you accept/reject post-acute care?
5. Which post-acute care model did you choose? Why? What factors did you consider?

### Study subjects and recruitment

The purpose of this study was to understand the factors influencing the choice of PAC services and models by stroke patients and their families, and the data provided by the research subjects was expected to be diverse and rich to help achieve the purpose of the study. Purposive sampling was adopted in the study, and the inclusion criteria for the study participants were as follows: (1) patients aged more than 20 years; (2) patients and family members of patients who were diagnosed with acute cerebral apoplexy within the past six months and have been treated in an advanced emergency responsibility hospital in northern, central, or southern Taiwan or in a moderate responsibility hospital with advanced acute treatment capacity for cerebral apoplexy; (3) stroke patients and family members of patients with moderate or moderate to severe functional impairment (modified Rankin scale of 2–5) at the time of the acute stroke; (4) stroke patients and family members of patients who had no cognitive impairment or mental illness and were able to answer the interview questions; and (5) stroke patients and family members who were willing to sign the interview consent form.

The research subjects for the qualitative interview were recruited mainly through the stroke treatment and care teams of the moderate and advanced emergency responsibility hospitals in northern, central, and southern Taiwan, which provided the subjects who met the research criteria. After the investigators confirmed that the subjects met the criteria, the subjects were contacted and the purpose as well as methods of the interview were described. If the subjects were willing to be interviewed, appointments were made on the interview time and place.

After continuous analysis by the investigators, no new ideas were identified in the newly collected data after the 21st respondent. Therefore, the recruitment of stroke patients was discontinued [24].

### Data collection

This study has obtained a clinical trial/research consent certificate from the ethics committee of the institutional review board of the Chang Gung Medical Foundation, and the review case number is 201900436B0C601. The researchers conducted in-depth interviews from 23 to 2019 to 16 December 2019. A semi-structured interview outline was used to conduct face-to-face interviews. First, the researchers introduced themselves to the interviewee and explained in detail the purpose of the study, the research interview process, and the rights of the study interviewee, while also assuring them that the research would comply with privacy regulations and ensure the privacy of personal data in accordance with the principles of confidentiality. After understanding the study purpose and related rights, the interviewee would sign the respondent consent form. Then, the interviewees were informed that the entire interview would be recorded and that if they do not want recordings, the investigators would respect that wish and record the interview content by hand. The interview and the recording could be interrupted at any time should the interviewee feel uncomfortable during the interview. The interview took approximately 25–40 min. The investigators entrusted the research assistants with the task of transcribing the interview recording files into a verbatim transcript within two days after each interview to establish the written data of the interview. All research related activities of the manuscript were performed in accordance with the Declaration of Helsinki.

### Data analysis

Content analysis was used to analyse the qualitative interview data in this study. The researchers carefully confirmed whether there were any errors in the verbatim transcripts of the interviews before the analysis. If any inconsistency with the original intended meaning was found or text was missing, the researchers made corrections after confirming the content of the audio file in person. In this study, the content analysis was mainly performed using the inductive approach and the data was divided into meaning units, condensed meaning units, codes, categories, and themes [25]. First, the researchers read the interview data repeatedly, i.e., the unit of analysis, and extracted paragraphs with the same statements in the text, which was the meaning unit. Then, the condensation of meaning units was done by a process of reducing the text while still preserving the core. The meaning units were subsequently coded according to the content of the material, and researchers labelled codes through assigning codes on the meaning units. After the coding process, all the codes were categorized. Finally, these different categories with related underlying meanings were grouped under the same theme [26].

## Results

### Respondent characteristics

During the study period from 23 to 2019 to 16 December 2019, 21 stroke patients (including eight females and thirteen males) and their family members were recruited from four different hospitals in northern, central, and southern Taiwan. Among them, there were 20 patients with ischemic stroke and one patient with haemorrhagic stroke, with a mean age of 65.6 years (range 33–92 years). The mean time to onset of stroke at the time of interview was nine weeks (range 3–23 weeks) (see Table 2 for details).

**Table 2:** Personal characteristics of all participants

NO	Age (years)	Gender	Admission MRS†	Discharged MRS	Educational level	Main caregiver	Hospital location and level	Stroke type	Onset of stroke (weeks)‡
1	61	Male	3	2	University	Spouse	Northern Academic medical center	Ischemic	13
2	76	Female	3	3	Elementary school	Child	Northern Academic medical center	Ischemic	6
3	60	Male	4	3	Elementary school	Spouse	Northern Academic medical center	Ischemic	2
4	62	Female	2	2	Senior high school	Spouse	Northern Academic medical center	Ischemic	15
5	55	Male	2	2	Senior high school	Spouse	Northern Academic medical center	Hemorrhage	10
6	52	Male	2	2	University	Spouse	Northern Academic medical center	Ischemic	2
7	55	Female	3	3	Senior high school	Spouse	Northern Academic medical center	Ischemic	3
8	78	Male	4	3	Elementary school	Child	Northern Academic medical center	Ischemic	20
9	62	Female	3	3	Senior high school	Spouse	Northern Academic medical center	Ischemic	11
10	83	Female	3	2	No formal education	Child	Northern Academic medical center	Ischemic	2
11	33	Female	3	4	University	Parents	Northern Academic medical center	Ischemic	11
12	80	Male	4	3	No formal education	Spouse	Northern Academic medical center	Ischemic	6
13	92	Male	4	2	No formal education	Child	Northern Academic medical center	Ischemic	11
14	76	Male	3	4	No formal education	Foreign care worker	Northern Regional Teaching	Ischemic	7
15	49	Male	4	3	Senior high school	Spouse	Northern Regional Teaching	Ischemic	23
16	75	Female	4	3	Elementary school	Child	Central Regional Teaching	Ischemic	3
17	75	Male	3	3	Junior high school	Family	Southern Academic medical center	Ischemic	12
18	57	Male	4	3	Senior high school	Spouse	Southern Academic medical center	Ischemic	10
19	81	Female	5	4	No formal education	Child	Southern Academic medical center	Ischemic	7
20	57	Male	2	2	Senior high school	Spouse	Southern Academic medical center	Ischemic	6
21	59	Male	2	2	Junior high school	Spouse	Southern Academic medical center	Ischemic	16

†MRS = Modified Rankin Scale, ‡ Time Calculation: stroke onset to interview

### Theme

**Fig. 1:**
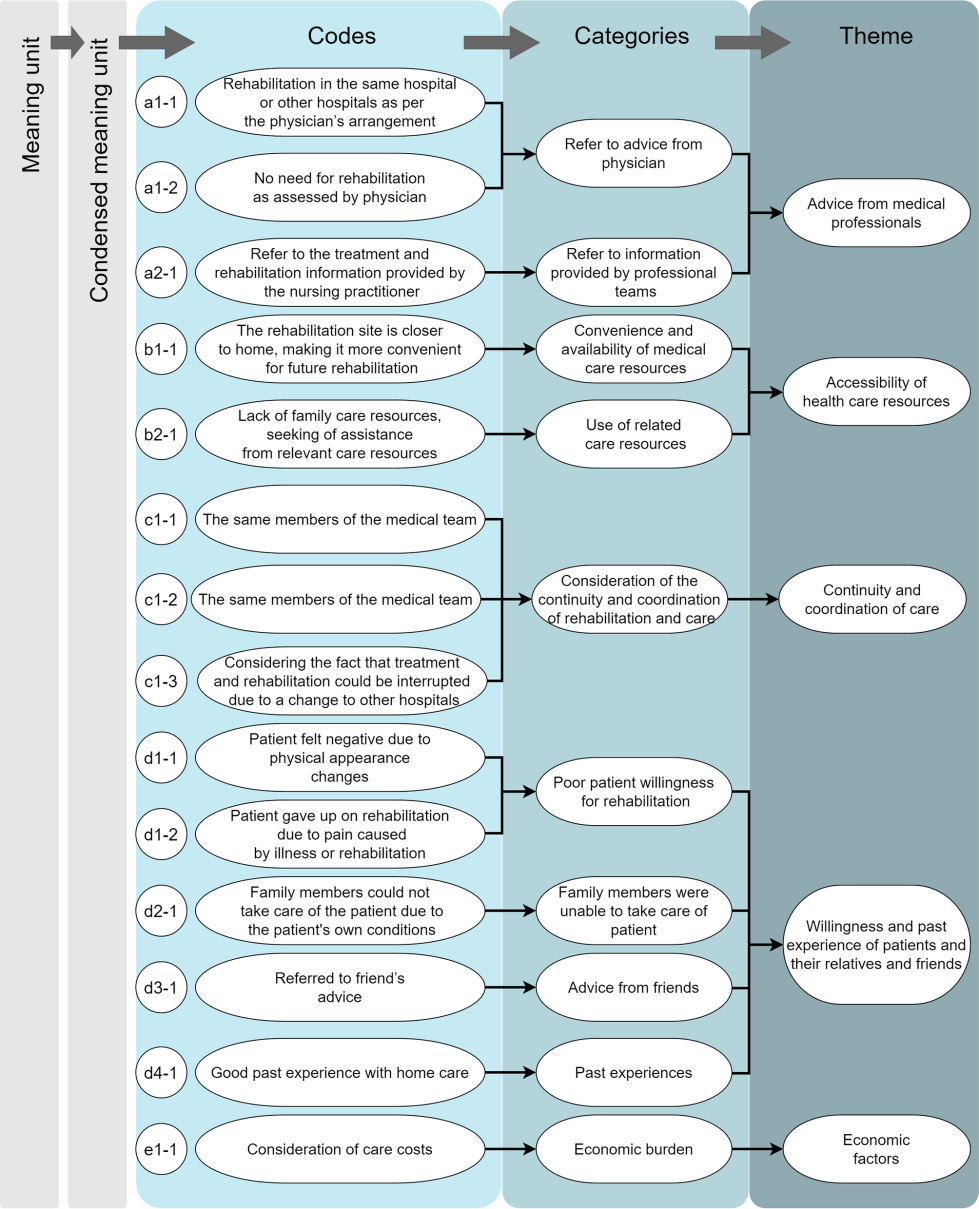
Codes, categories and theme, and code of factors influencing the choice of PAC model for stroke patients and their families

The results of analysis of the interviews of 21 stroke patients and their family members pointed out that there were five main factors affecting the patients’ choice of PAC models (as detailed in Table 3; Fig. 1), namely (1) the advice of medical professionals, (2) the accessibility of health care resources, (3) the continuity and coordination of care, (4) the willingness and past experience of patients and their relatives and friends, and (5) economic factors. The main factors are described below.

**Table 3:** Meaning unit and Condensed meaning unit

Meaning unit	Condensed meaning unit
The doctor said that I had a nerve injury, so they put pressure on my brain first. Then after about three days, when there was no bleeding, they arranged rehabilitation for me to see how far I could go. So, I was scheduled to undergo rehabilitation in the hospital. As they were all known members of the medical team, I accepted the doctor’s advice. I was in hospital for probably about a week. (N05)	They would arrange rehabilitation for me… So, rehabilitation was arranged in the hospital. I was in hospital for probably about a week. (a1-1)
We followed the doctor’s advice and stayed in the hospital for treatment and rehabilitation. The rehabilitation programme included functional therapy, speech therapy, and acupuncture. So, we stayed in the hospital for three months. We hope to receive complete rehabilitation in order to have a smooth recovery. (N06)	We followed the doctor’s advice and stayed in the same hospital for treatment and rehabilitation…… We stayed in the hospital for three months. (a1-1)
At about the third month of hospitalisation, they suggested that I go to another hospital or clinic for rehabilitation. (N09)	At the third month of hospitalisation… I was advised to go to other hospitals or clinics for rehabilitation. (a1-1)
At that time, the stroke doctor helped me consult a physiatrist. But the physiatrist said that rehabilitation would not be effective for me, so I did not continue rehabilitation. (N11)	The physiatrist said that rehabilitation would not be effective, so I did not receive rehabilitation. (a1-2)
Because I have had 3–4 strokes, the doctor suggested maintaining the status quo. However, when I had a stroke before, the doctor suggested that I go to the rehabilitation centre in Xinzhuang for rehabilitation. (N02)	I have had 3–4 strokes… The doctor suggested maintaining the status quo. However, when I had a stroke before, the doctor suggested that I go to the rehabilitation centre in Xinzhuang for rehabilitation. (a1-2)
When we were preparing to be discharged from the hospital, the nurse gave us a form with information on rehabilitation medical institutions for our reference, and we made choices based on the list of rehabilitation hospitals in that form. (N04)	The nurse gave us a form with information on rehabilitation medical institutions for our reference, and we made choices based on the list of rehabilitation hospitals in that form. (a2-1)
Before I was discharged, the hospital gave me a piece of paper with information about rehabilitation hospitals for reference, and I also searched the Internet for rehabilitation hospitals near my home… I am currently on rehabilitation at a clinic near my home. At that time, the doctor told me that I could come back to the original treatment hospital for outpatient rehabilitation, but it was too far. I wanted to rehabilitate at the clinic near us. It would be more convenient for me to be closer to home. So, I discussed it with the doctor. (N04)	The hospital gave me a piece of paper with information about rehabilitation hospitals for reference… I also searched the Internet for rehabilitation hospitals near my home… The doctor told me that I could come back to the original treatment hospital for outpatient rehabilitation, but it was too far. Rehabilitation at the clinic near us would be more convenient for me. (b1-1)
After staying for more than ten days, we considered going to the Miners’ Hospital as it was closer to our home because he needed to be on rehabilitation for a long time. If he received rehabilitation in the hospital in Keelung… the distance would be inconvenient. So, we discussed with the doctor saying that we wanted to go to the Miners’ Hospital for rehabilitation. (N15)	Because he needed to be on rehabilitation for a long time, the Miners’ Hospital was seen as more convenient as it was closer to our home. If he received rehabilitation in the hospital in Keelung…the distance would be inconvenient. (b1-1)
The hospital actually suggested that we go to a nearby hospital, but we did not have enough people to take care of him. He could not take care of himself, having trouble urinating and eating, so I had to apply for resources for foreign workers and long-term care. (N08)	The hospital actually suggested that we go to a nearby hospital… but we did not have enough people to take care of him… He could not take care of himself, having trouble urinating and eating, so I had to apply for resources for foreign workers and long-term care. (b2-1)
He cannot receive any treatment at the moment, because we siblings have to go to work. Only our mother can help take care of him… The doctor actually suggested that we go to a nearby hospital for rehabilitation, but we lacked the manpower to take care of him…. So, we applied for long-term care and foreign nursing care. (N10)	He cannot receive any treatment at the moment, because we siblings have to go to work. Only our mother can help take care of him… The doctor actually suggested that we go to a nearby hospital for rehabilitation, but we could not take care of him…. So, we applied for long-term care and foreign nursing care. (b2-1)
I was scheduled to undergo rehabilitation in the hospital. As they were all known members of the medical team, I accepted the doctor’s advice. I was in hospital for probably about a week. (N05)	As they were all known members of the medical team, I accepted the doctor’s advice. (c1-1)
we stayed in the hospital for three months. We hope to receive complete rehabilitation in order to have a smooth recovery. (N06)	We hope to receive complete rehabilitation in order to have a smooth recovery. (c1-2)
My husband was afraid that no one would urge me to exercise when I was at home… If I participated in the PAC rehabilitation programme, there would be specialised stroke physicians, physiatrists, rehabilitation therapists, and ward nurses to assist me in rehabilitation. The nurse would also guide me to do some stretching exercises on the hospital bed, etc… If I transferred to another hospital for rehabilitation, I would worry about whether the coordination would be as smooth. During inpatient rehabilitation, I also saw some patients who were more serious than me. But after working hard on rehabilitation, some of them recovered very well and could walk on their own. (N09)	In the PAC rehabilitation programme I participated in, there were specialised stroke physicians, physiatrists, rehabilitation therapists, and ward nurses to assist me in rehabilitation. The nurse would also guide me to do some stretching exercises on the hospital bed, etc… If I transferred to another hospital for rehabilitation, I would worry about whether the coordination would be as smooth. (c1-3)
When he had his first stroke, he went back to the hospital for rehabilitation, which lasted for more than ten years. But ever since the urinary catheter was placed, he did not like to walk outside or go to the hospital. So, no rehabilitation was arranged for him when he had a stroke this time. (N14)	When he had his first stroke, he was on rehabilitation for more than ten years… But ever since the urinary catheter was placed, he did not like to walk outside or go to the hospital. So, no rehabilitation was arranged for him. (d1-1)
We went to a clinic near our home for rehabilitation earlier. But because she was afraid of pain, she stopped going after a while. The main reason was that she was not proactive. Later, she developed backache, so she did not want to go even more and became even more resistant. As she did not like it, we did not want to force her. (N10)	We went to a clinic near our home for rehabilitation earlier. But because she was afraid of pain, she stopped going after a while… The main reason was the negative attitude. Later, she developed backache, so she did not want to go even more and became even more resistant…(d1-2)
The doctor told us that after being discharged from the hospital, we could choose to go to a medical institution or take care of him at home. We wanted to take him home and take care of her, but in reality, we could not do so because she still required suctioning of the thick phlegm and had to rely on oxygen after being discharged from the hospital. No one dared to pump the phlegm out, so we had to let him go to a nursing home first. (N19)	The doctor said that after being discharged from the hospital, we could choose to go to a medical institution or take care of him at home. We wanted to take her home and take care of him, but in reality, we could not do so because she had to rely on oxygen when she was discharged from the hospital. No one dared to pump the phlegm out, so we had to let her go to a nursing home first. (d2-1)
It was not long after I was hospitalised, I went to Taipei Wanhua Hospital for hyperbaric oxygen therapy at the suggestion of a friend. (N01)	Received hyperbaric oxygen therapy in Taipei Wanhua Hospital at the suggestion of a friend. (d3-1)
This was my first stroke. I stayed in hospital for about two weeks, and one week after I was hospitalised, a rehabilitator came to the ward for rehabilitation and helped with stretching exercises of the hands and feet. After I was discharged from the hospital, I chose the current rehabilitation hospital mainly because of the environment, equipment, and recommendations from friends. (N16)	After I was discharged from the hospital, I chose the current rehabilitation hospital mainly because of the environment, equipment, and recommendations from friends. (d3-1)
We have chosen home care for several years. Somebody would come to our home to help with stretching exercises every day. Every month, home care would send a doctor to our home or give us a call to check on dad’s condition. Is there any need to increase or decrease rehabilitation measures?… We would also discuss with the doctors in the hospital… We felt that home care was very good. So, when the hospital wanted to assist us in applying for home care, we agreed. (N03)	We have chosen home care for several years… Somebody would come to our home to help with stretching exercises every day…….We felt that home care was very good. So, when the hospital wanted to assist us in applying for home care, we agreed. (d4-1)
Everyone has to go to work on weekdays. It is more convenient for us to choose home care, and the burden is lighter than that of foreign care. (N03)	Everyone has to go to work on weekdays. It is more convenient for us to choose home care, and the burden is lighter than that of foreign care. (e1-1)
In addition to choosing the kind of care that was the best for him, what was also important was that the cost of care should be affordable for us. During hospitalisation earlier, we hired a Taiwanese care worker for NT$ 2,000 a day. We stayed in the hospital for more than ten days and spent more than NT$ 30,000. (N08)	In addition to choosing the kind of care that was the best for him, what was also important was that the cost of care should be affordable for us. (e1-1)

#### Advice of medical professionals

It was found from the interview results that among the 21 respondents, five patients would choose to accept the arrangement made by the acute care physician for rehabilitation at the same hospital or other hospitals. Information on post-discharge treatment and rehabilitation provided by medical institutions and relevant medical professionals would also be referred to. Patients or families may also choose not to receive follow-up rehabilitation when a specialist or physiatrist assesses that there is no need for rehabilitation. However, the choices were inhibited by medical urgency and need and limited to those listed by professionals, hence they were limited under particular conditions or contexts.

“When we were preparing to be discharged from the hospital, the nurse gave us a form with information on rehabilitation medical institutions for our reference, and we made choices based on the list of rehabilitation hospitals in that form.” (N04).

The doctor said that I had a nerve injury, so they put pressure on my brain first. Then after about three days, when there was no bleeding, they arranged rehabilitation for me to see how far I could go. So, I was scheduled to undergo rehabilitation in the hospital. I was in hospital for probably about a week. (N05)

“We followed the doctor’s advice and stayed in the hospital for treatment and rehabilitation. The rehabilitation programme included functional therapy, speech therapy, and acupuncture. So, we stayed in the hospital for three months.” (N06).

“At that time, the stroke doctor helped me consult a physiatrist. But the physiatrist said that rehabilitation would not be effective for me, so I did not continue rehabilitation.” (N11).

#### Accessibility of health care resources

After the acute inpatient care phase, stroke patients usually entered a rehabilitation phase of 3 months, depending on the degree of disability, so they carried out quite complex calculations based upon the logistics of attending a medical institution far from home for PAC. Among the interviewed cases, two patients would consider the accessibility of medical resources as well as the convenience of medical treatment in the future when choosing the PAC models. They would choose a medical institution closer to their homes to receive PAC.

Before I was discharged from the hospital, the hospital gave me a piece of paper with information about rehabilitation hospitals for reference, and I also searched the Internet for rehabilitation hospitals near my home… I am currently on rehabilitation at a clinic near my home. At that time, the doctor told me that I could come back to the original treatment hospital for outpatient rehabilitation. I wanted to rehabilitate at a clinic near us. It would be more convenient for me to be closer to home. So, I discussed it with the doctor. (NO4)

After staying for about ten days, we considered going to the Miners’ Hospital as it was closer to our home because he needs to be on rehabilitation for a long time. If he receives rehabilitation in the hospital in Keelung… the distance would be inconvenient. So, we discussed with the doctor saying that we wanted to go to the Miners’ Hospital for rehabilitation. (N15)

In addition to the accessibility of medical care resources, the accessibility of other health care resources including foreign nursing care and long-term care also affected the choice of PAC services and models. The other two patients would consider the family structure and the burden on family members while choosing a method so that the impact on the daily work and life of family members is less, such as applying for foreign nursing care, long-term care, and home care models.

The hospital actually suggested that we go to a nearby hospital, but we could not take care of him. He could not take care of himself, having trouble urinating and eating, so I had to apply for resources for foreign workers and long-term care. (N08)

He cannot receive any treatment at the moment, because we siblings have to go to work. Only our mother can help take care of him, but my mom is not in very good health now. The doctor actually suggested that we go to a nearby hospital for rehabilitation, but we lack the manpower to take care of him…. So, we applied for long-term care and foreign nursing care, which made it more convenient for us to go to work. It was also easier on our mom. Only after we adapt to the foreign nursing care will we take our father to the hospital for rehabilitation. (NO10)

#### Continuity and coordination of care

Some patients believed that staying in the original acute treatment hospital for follow-up rehabilitation allowed the patient to obtain more medical resources to implement a complete and continuous rehabilitation plan, and the same healthcare providers had a better understanding of the patient’s past medical history or patient preferences, so receiving care continuity and coordination was associated with better care outcomes. Therefore, the patient participated in the inpatient rehabilitation of the quality PAC pilot programme in the hospital. The patient wished to recover the functional damages caused by stroke through an intensive and regular rehabilitation programme.

I was scheduled to undergo rehabilitation in the hospital. As they were all known members of the medical team, I accepted the doctor’s advice. I was in hospital for probably about a week. (N05)

we stayed in the hospital for three months. We hope to receive complete rehabilitation in order to have a smooth recovery. (N06)

If I participated in the PAC rehabilitation programme, there would be specialised stroke physicians, physiatrists, rehabilitation therapists, and ward nurses to assist me in rehabilitation. The nurse would also guide me to do some stretching exercises on the hospital bed, etc… If I transfer to another hospital for rehabilitation, I will worry whether the coordination will be as smooth. During the inpatient rehabilitation, I also saw some patients who were more serious than me. But after working hard on rehabilitation, some of them recovered very well and could walk on their own. (NO9)

#### Willingness and past experience of patients and their relatives and friends

Some patients only took into account their wishes or the past post-acute care experiences of them and their relatives and friends and overlooked the need for more appropriate treatment or more active rehabilitation opportunities at this time. There were three patients who made different choices according to their own or their family members’ willingness. For example, the placement of nasogastric tube and urinary catheter due to functional impairment as a result of stroke affected the patients’ willingness for rehabilitation. In cases where the patient was unable to bear the physical discomfort, the willingness for rehabilitation was reduced. In cases where family members were unable to care for serious stroke patients, home nursing was chosen as the post-discharge care model.

We went to a clinic near our home for rehabilitation earlier. But she stopped going after a while because she was afraid of pain. The main reason was that she was not proactive. Later, she developed backache, so she did not want to go even more and became even more resistant. As she did not like it, we did not want to force her. (N10)

When he had his first stroke, he went back to the hospital for rehabilitation, which lasted for more than ten years. But ever since the urinary catheter was placed, he did not like to walk outside or go to the hospital. So, no rehabilitation was arranged for him when he had a stroke this time. (N14)

The doctor told us that after being discharged from the hospital, we could choose to go to a medical institution or take care of her at home. We wanted to take her home and take care of him, but in reality, we could not do so because she still required suctioning of the thick phlegm and had to rely on oxygen after being discharged from the hospital. No one dared to pump the phlegm out, so we had to let him go to a nursing home first. (N19)

There were three other patients who chose the current PAC model based on their past experiences with PAC. For example, case NO3 had a good experience with home care in the past, so they continued using the same model this time. The other two patients referred to the advice and experience of relatives and friends.

“It was not long after I was hospitalised, I went to Taipei Wanhua Hospital for hyperbaric oxygen therapy at the suggestion of a friend.” (N01).

This was my first stroke. I stayed in hospital for about two weeks. One week after I was hospitalised, a rehabilitator came to the ward for rehabilitation and helped with stretching exercises of the hands and feet. After I was discharged from the hospital, I chose the current rehabilitation hospital mainly because of the environment, equipment, and recommendations from friends. (N16)

We have chosen home care for several years. Somebody would come to our home to help with stretching exercises every day. Every month, home care would send a doctor to our home or give us a call to check on dad’s condition. Is there any need to increase or decrease rehabilitation measures? We would also discuss with the doctors in the hospital… We felt that home care was very good. So, when the hospital wanted to assist us in applying for home care, we agreed. (N03)

#### Economic factors

The cost of care varies depending on the PAC models chosen, and if a family member takes days off to care for a stroke patient, the loss of salary due to absence was also taken into account, so the cost of care was a heavy burden for the family. Two patients said that although financial burden was not their only consideration, it would certainly affect their choice of the PAC models. They would evaluate and choose a model that was helpful to the patient’s functional recovery and also involved affordable health care costs.

“Everyone has to go to work on weekdays. It is more convenient for us to choose home care, and the burden is lighter than that of foreign nursing care.” (N03).

In addition to choosing the kind of care that was the best for him, what was also important was that the cost of care should be affordable for us. During hospitalisation earlier, we hired a Taiwanese care worker for NT$ 2,000 a day. We stayed in the hospital for more than ten days and spent more than NT$ 30,000. (N08)

## Discussion

Based on the results of the in-depth interviews, this study identified five main factors that affect the choice of PAC models among stroke patients and their families, which are the advice of medical professionals, the accessibility of health care resources, the continuity and coordination of care, the willingness and past experience of patients and their relatives and friends, and economic factors.

Patients and their families refer to or directly follow the advice of medical professionals in choosing the PAC models, which was consistent with previous research findings. The professional attitudes of different medical providers and the habits of providing medical care affect the patient’s choice of PAC models [16]. A previous qualitative study of stroke patients in the United States found that more than half of the patients did not participate in the selection of their PAC models, and that approximately two-fifths of stroke patients did not participate in setting rehabilitation goals. However, all these patients trusted the medical care decisions made by their physicians and most patients were satisfied with their rehabilitation models [27].

Patients and their families also consider the accessibility of health care resources when choosing the PAC model, which was also consistent with previous studies [19, 28, 29]. According to the results of this study, medical professionals provide advice or relevant reference information for choosing the PAC models. Moreover, some patients seek relevant health care resources, such as applying for home care and long-term care resources, in order to reduce the burden on the family members in caring for the patient and to reduce the commute time between their home and hospital. Some patients consider the convenience of receiving medical treatment in the future and choose medical institutions closer to their homes for PAC services. It is worth noting that although the distance between the location of the PAC service and patients’ home is an important factor affecting these choices, it might also be so because the patients and their families do not have access to other information on the quality of medical care, such as the quality monitoring reports published by technical nursing institutions [19]. Another study showed that even when information on the location of PAC institutions and the quality of medical care was provided, many patients still chose institutions closer to their homes for PAC services, while only a small number of patients and their families were willing to choose distant PAC institutions for better quality of care [28, 29].

The results of this study showed that the continuity and coordination of care were also contributing factors. This finding may support previous study results on the importance and relevance of continuity and consistency of care in improving the quality of medical care [17, 18]. Patients with cerebral apoplexy would like a complete and consistent care plan, and therefore choose to stay in the original acute treatment hospital for post-acute inpatient rehabilitation. According to the results of a qualitative study on the continuity and consistency of outpatient PAC and inpatient rehabilitation, many patients receiving outpatient rehabilitation considered outpatient rehabilitation to be discontinuous [17], while most patients receiving inpatient rehabilitation believed that medical care information, treatment management, and the relationship with medical providers were the main factors affecting the continuity and coordination of care [18]. This result demonstrates the importance of continuity of care for patients and their families, as well as the hope that medical providers and various medical teams would establish consistent care models.

This study also pointed out that the willingness and past experiences of patients and their relatives and friends were also contributing factors. Previous studies have also reported the same results [19, 20]. The previous experiences of patients and their relatives and friends as well as their familiarity with the PAC models affected the choice of PAC services [19, 20]. Patients trusted the judgement and experience of those they knew, and patients may once again choose a nursing technology institution that they had previously used even if the previous experience had been unsatisfactory [19]. Patient willingness was also an important factor for the selection of PAC models. In a study on the preference of inpatients with cerebral apoplexy for post-acute care and rehabilitation, it was found that even if aggressive inpatient rehabilitation might improve functional outcomes, nearly 85% of patients still wished to rehabilitate at home through the home care or other models rather than choose inpatient rehabilitation or nursing institutions [30].

Economic factors also affected the choice of patients and their families. In the past, studies have pointed out that patients refused to accept PAC because they could not afford health care or insurance costs [20]. In this study, the researchers found that although economic factors were not the only consideration for patients and their families in choosing PAC models, they would still choose a care model with a lower burden of care costs. However, previous study results have pointed out that health care costs would affect the choice of PAC models among patients, primary caregivers, and even clinical medical staff, thereby affecting the care quality and safety of patients [31]. Therefore, providing patients and caregivers with explanations and information on the cost of PAC models may help patients choose the appropriate care model.

In recent years, the incidence of stroke and related mortality have been on the decline, which may be attributed to the control of risk factors for stroke, including control of hypertension, diabetes, and dyslipidaemia, treatment of atrial fibrillation, and decreased smoking rates. In addition, the acute care of cerebral apoplexy and PAC have also played an important role [32–34]. For patients with cerebral apoplexy, early rehabilitation and acceptance of PAC may significantly reduce the risk of disability and death [5, 6, 10–12]. Before the implementation of the post-acute care pilot programme by the National Health Insurance Administration, stroke patients in Taiwan generally stayed in acute treatment hospitals for follow-up medical rehabilitation services. According to a 2012 study by Wu et al. [35] that sought to assess the needs of PAC in Taiwan, patients with cerebral apoplexy in Taiwan often replaced PAC with rehospitalisation or ultra-long-term hospitalisation. The implementation of PAC may reduce the number of days of acute hospitalisation, thereby helping realise the efficient use of medical resources. Therefore, this study explored the choice of the PAC models for stroke patients from the perspectives of patients and their families and identified five main influencing factors that can inform the decision-making of policy makers, clinical medical professionals, as well as patients and their families, which in turn would promote communication, implement shared decision-making in medical care, improve doctor-patient relationship, and ultimately improve care outcomes.

### Limitations

Although the respondents of the study were 21 stroke patients and their family members from four hospitals in northern, central, and southern Taiwan, and detailed information was provided on the factors influencing their choice of the PAC models, the study results were only the opinions of the patients and their families and cannot represent the experiences of non-respondents since most of the cases received care in northern hospitals; moreover, almost all cases involved ischemic stroke, with haemorrhagic stroke occurring in only one case. In this study, in-depth, face-to-face interviews were conducted in the outpatient area of ​​the hospital. Although none of the interviewees stopped the interview due to privacy concerns, the outpatient waiting area of ​​the hospital was an open space, and the interviewees may have been affected by the surrounding people and environment.

## Conclusion and suggestions

The advice of medical professionals, the accessibility of health care resources, the continuity and coordination of care, the willingness and past experience of patients and their relatives and friends, and economic factors were five important factors that affected the selection of PAC models for stroke patients. Follow-up studies should extensively explore the impacts on different cases based on the five main factors identified in this study. Future policy makers should establish medical resources and care models that meet patient requirements based on the views of patients and their families. In clinical practice, in addition to providing professional medical advice and objective medical information to patients and family members, clinical medical professionals should also understand and align their suggestions with patients’ preferences and values ​​to help stroke patients choose the appropriate acute care models and improve the quality of care.

## Data Availability

The data that support the fndings of this study are available on request from the corresponding author. But the data are not publicly available due to example to protect study participant privacy.

## Abbreviations

PAC: post-acute care
IRH: inpatient rehabilitation hospital
OP: outpatient
HH: home health
CMS: the Centers for Medicare and Medicaid Services
MRS: Modified Rankin Scale

## References

[1] Murray CJ, Lopez AD (2013). Measuring the global burden of disease. N Engl J Med.

[2] Feigin VL, Forouzanfar MH, Krishnamurthi R, Mensah GA, Connor M, Bennett DA, Moran AE, Sacco RL, Anderson L, Truelsen T (2014). Global and regional burden of stroke during 1990–2010: findings from the global burden of Disease Study 2010. Lancet (London England).

[3] Mozaffarian D, Benjamin EJ, Go AS, Arnett DK, Blaha MJ, Cushman M, de Ferranti S, Despres JP, Fullerton HJ, Howard VJ (2015). Heart disease and stroke statistics–2015 update: a report from the American Heart Association. Circulation.

[4] Bettger JP, Thomas L, Liang L, Xian Y, Bushnell CD, Saver JL, Fonarow GC, Peterson ED. Hospital Variation in Functional Recovery After Stroke.Circulation Cardiovascular quality and outcomes2017, 10(1). 10.1161/CIRCOUTCOMES.115.002391 28096203

[5] Hankey GJ, Spiesser J, Hakimi Z, Bego G, Carita P, Gabriel S (2007). Rate, degree, and predictors of recovery from disability following ischemic stroke. Neurology.

[6] Hsieh CY, Huang HC, Wu DP, Li CY, Chiu MJ, Sung SF. Impact of rehabilitation intensity on mortality risk after stroke.Archives of physical medicine and rehabilitation2017. 10.1016/j.apmr.2017.10.011 29108967

[7] Buntin MB, Garten AD, Paddock S, Saliba D, Totten M, Escarce JJ (2005). How much is postacute care use affected by its availability?. Health Serv Res.

[8] Buntin MB (2007). Access to postacute rehabilitation. Arch Phys Med Rehabil.

[9] National Health Insurance Administration of Taiwan. *Integrated post-acute care plan*. Available at: [https://www.nhi.gov.tw/Content_List.aspx?n=5A0BB383D955741C&topn=D39E2B72B0BDFA15]

[10] Buntin MB, Colla CH, Deb P, Sood N, Escarce JJ (2010). Medicare spending and outcomes after postacute care for stroke and hip fracture. Med Care.

[11] Wang H, Sandel ME, Terdiman J, Armstrong MA, Klatsky A, Camicia M, Sidney S (2011). Postacute care and ischemic stroke mortality: findings from an integrated health care system in northern California. PM R.

[12] Lai CL, Tsai MM, Luo JY, Liao WC, Hsu PS, Chen HY (2017). Post-acute care for stroke - a retrospective cohort study in Taiwan. Patient Prefer Adherence.

[13] Topaz M, Kang Y, Holland DE, Ohta B, Rickard K, Bowles KH (2015). Higher 30-day and 60-day readmissions among patients who refuse post acute care services. Am J Manag Care.

[14] Magdon-Ismail Z, Sicklick A, Hedeman R, Bettger JP, Stein J (2016). Selection of Postacute Stroke Rehabilitation Facilities: a Survey of Discharge Planners from the Northeast Cerebrovascular Consortium (NECC) region. Med (Baltim).

[15] Medicare Cf, Services M. Medicare and Medicaid programs; revisions to requirements for discharge planning for hospitals, critical access hospitals, and home health agencies. *Federal Register [serial on the Internet]* 2019.

[16] Ottenbacher KJ, Graham JE (2007). The state-of-the-science: access to postacute care rehabilitation services. A review. Arch Phys Med Rehabil.

[17] Medina-Mirapeix F, Oliveira-Sousa S, Sobral-Ferreira M, Del Bano-Aledo ME, Escolar-Reina P, Montilla-Herrador J, Collins SM (2011). Continuity of rehabilitation services in post-acute care from the ambulatory outpatients’ perspective: a qualitative study. J Rehabil Med.

[18] Medina-Mirapeix F, Oliveira-Sousa SL, Escolar-Reina P, Sobral-Ferreira M, Lillo-Navarro MC, Collins SM (2017). Continuity of care in hospital rehabilitation services: a qualitative insight from inpatients’ experience. Braz J Phys Ther.

[19] Gadbois EA, Tyler DA, Mor V (2017). Selecting a skilled nursing facility for postacute care: individual and family perspectives. J Am Geriatr Soc.

[20] Sefcik JS, Ritter AZ, Flores EJ, Nock RH, Chase JD, Bradway C, Potashnik S, Bowles KH (2017). Why older adults may decline offers of post-acute care services: a qualitative descriptive study. Geriatr Nurs.

[21] Conducting in-depth interviews. : A guide for designing and conducting in-depth interviews for evaluation input [https://d1wqtxts1xzle7.cloudfront.net/33661461/m_e_tool_series_indepth_interviews-with-cover-page-v2.pdf?Expires=1647573598&Signature=EeMYhYDFmZMBvShVO9VGV8VeB90C~jbmlbQGlb4LI7P22giySbYccEk8NVEBbCO86NGx~wGSAA7g4dNQXWc2Me18tlkntpn~c7vfTYfmQhmoGQHXhT8hnn1UABeV9nRY6IgW2Xr63Zz5x~BRYcJUNjIhSfhCYX5aSfV3MK5lrhwbgSdMPE5H~0It1LJVsGYYUeKR5JGLkLm4Aw06xy8TRx9o65iktAIr~~c9H-NNfX~uFY450KCkaLGsspRYZq38aCjG7LHbcbmwfCbsW~j1TiR-mX5YSTrQfkupYu9sEDlp7ya7BYtZPHNK9lXEbOe3DVWIXxFtnUnEDIRJeqXpCA__&Key-Pair-Id=APKAJLOHF5GGSLRBV4ZA]

[22] Milena ZR, Dainora G, Alin S (2008). Qualitative research methods: a comparison between focus-group and in-depth interview. Annals of the University of Oradea Economic Science Series.

[23] McIntosh MJ, Morse JM (2015). Situating and constructing diversity in semi-structured interviews. Global qualitative nursing research.

[24] Hennink MM, Kaiser BN, Marconi VC (2017). Code saturation versus meaning saturation: how many interviews are enough?. Qual Health Res.

[25] Graneheim UH, Lundman B (2004). Qualitative content analysis in nursing research: concepts, procedures and measures to achieve trustworthiness. Nurse Educ Today.

[26] Elo S, Kyngäs H (2008). The qualitative content analysis process. J Adv Nurs.

[27] Krishnan S, Hay CC, Pappadis MR, Deutsch A, Reistetter TA (2019). Stroke survivors’ perspectives on Post-Acute Rehabilitation Options, Goals, satisfaction, and transition to Home. J Neurol Phys Ther.

[28] Pesis-Katz I, Phelps CE, Temkin-Greener H, Spector WD, Veazie P, Mukamel DB (2013). Making difficult decisions: the role of quality of care in choosing a nursing home. Am J Public Health.

[29] Hefele JG, Acevedo A, Nsiah-Jefferson L, Bishop C, Abbas Y, Damien E, Ramos C (2016). Choosing a nursing home: what do consumers want to know, and do preferences vary across Race/Ethnicity?. Health Serv Res.

[30] Gregory P, Edwards L, Faurot K, Williams SW, Felix AC (2015). Patient preferences for stroke rehabilitation. Top Stroke Rehabil.

[31] Ayele R, Jones J, Ladebue A, Lawrence E, Valverde P, Leonard C, Cumbler E, Allyn R, Burke RE (2019). Perceived costs of Care Influence Post-Acute Care Choices by Clinicians, Patients, and caregivers. J Am Geriatr Soc.

[32] Koton S, Schneider AL, Rosamond WD, Shahar E, Sang Y, Gottesman RF, Coresh J (2014). Stroke incidence and mortality trends in US communities, 1987 to 2011. JAMA.

[33] Lee M, Wu YL, Ovbiagele B (2016). Trends in Incident and recurrent rates of first-ever ischemic stroke in Taiwan between 2000 and 2011. J stroke.

[34] Koton S, Sang Y, Schneider ALC, Rosamond WD, Gottesman RF, Coresh J (2020). Trends in Stroke Incidence Rates in older US adults: an Update from the atherosclerosis risk in Communities (ARIC) Cohort Study. JAMA Neurol.

[35] Kuan-Ying W, Shiao-Chi W, Hung Y-N, Chun-Chen W, Li-Chan L, Han-Hwa H (2012). The need for post-acute care for stroke patients in Taiwan. Taiwan J Public Health.

